# What needs to be done to control the spread of Middle East respiratory syndrome coronavirus?

**DOI:** 10.2217/fvl.15.20

**Published:** 2015-05-29

**Authors:** Michael Edelstein, David L Heymann

**Affiliations:** 1 Center on Global Health Security, The Royal Institute of International Affairs, Chatham House, 10 St James's Square, London, SW1Y 4LE, UK

**Keywords:** coronavirus, disease outbreak, emergency preparedness, surveillance

## Abstract

Up to November 2014, Middle East respiratory syndrome coronavirus (MERS-CoV) has infected 935 individuals and killed 371, all originating in or with links to the Middle East. The mechanisms of transmission of the disease are not fully understood, but MERS-CoV seems to sustain itself in the human population through repeated re-introduction from a camel reservoir and is able to cause nosocomial outbreaks. The risk of a global spread of MERS-CoV is low. Epidemiological, serological and phylogenetic research, combined with one health surveillance, dynamic case definitions, active case finding, rigorous infection control, culturally sensitive risk communication and a continuous re-evaluation of new evidence will enable to better understand the disease, limit its spread and quantify its risk in order to better prepare for a hypothetical spread.

**Figure 1.**
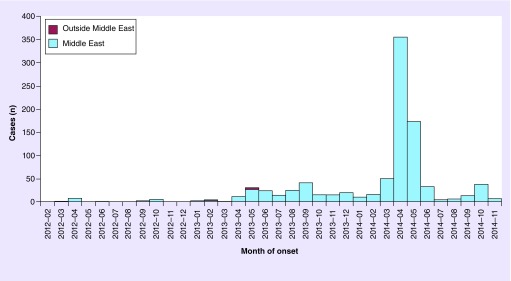
**Distribution of confirmed cases of MERS-CoV by first available date and place of probable infection, March 2012–November 2014 (n = 932).** Adapted with permission from the European Centre for Disease Prevention and Control.

## Background

The first human infection with Middle East Respiratory syndrome coronavirus (MERS-CoV) was reported in Saudi Arabia in 2012. Up to 7 November 2014, there have been 935 laboratory-confirmed MERS-CoV human infections, including 371 deaths, in 23 countries [1] (Figure 1). All cases have occurred in individuals in the Middle East, or in persons with recent travel to the Middle East, or who had contact with a person who had traveled to the Middle East [2]. Two thirds of all reported cases have been in males, and the outbreak peaked in April 2014 [2]. Since then, the number of reported cases has been decreasing, and human infections now appear to be sporadic (Figure 1).

Infection with MERS-CoV generally presents as an influenza-like illness. Typically, MERS rapidly progresses to viral pneumonia about a week after the onset of the infection [3]. Individuals with underlying chronic conditions or immunosuppression are at higher risk of severe disease [4]. MERS-CoV infection has mortality rate of 35–50% [5]. There is a lack of proven effective therapies and possible therapeutic are based on therapies used for SARS coronavirus, a phylogenetically related virus [6]. MERS is caused by a newly identified β-coronavirus, lineage C [7]. MERS-CoV genomes are phylogenetically classified into two clades, clade A and B clusters [8]. The viral genomes detected in the earliest human infections (clade A) are genetically distinct from the others (clade B). Taxonomically, MERS-CoV belongs to the same viral species as the African *Neoromicia capensis* bat coronavirus (NeoCoV) [9] with which it shares 85% of its genome at the nucleotide level [9] and up to 95% homology for genes encoding structural proteins [9]. Although infections with MERS-CoV and SARS coronavirus have similar clinical presentations, the two viruses belong to different lineages [10]. While the SARS coronavirus can be aerosolized [11] and sustained airborne transmission is possible [11,12], MERS-CoV human-to-human transmission has so far been limited and unsustained [13]. Recent camel and human MERS-CoV genetic sequences show no significant mutations compared with previous sequences from this outbreak [2], and the virus does not appear to have adapted toward sustained human-to-human transmission [14]. The current patterns of MERS-CoV transmission suggest repeated introduction of the virus in the human population with limited, unsustained human-to human transmission, mainly in hospitals, and in close family members [2], both of which are self-limiting because of the low reproductive number of the virus [14].

Camels are thought to be an important reservoir for MERS-CoV. Anti-MERS-CoV antibodies have been detected in samples that have been collected from dromedary camels in Saudi Arabia since 1992 [15] and the genomes of MERS-CoV viruses isolated from camels and humans are similar [16]. Phylogenetic data suggest that camels act as sources of virus for humans rather than vice versa [9]. In Egypt, over 90% of camels had antibody to MERS-CoV [17] and 3.6% were hosting the virus at the time of screening [7], suggesting that it is commonly circulating in the camel population. The virus has not been isolated from other mammal species tested [17]. Strengthening the hypothesis that camels infect humans, MERS-CoV with an identical sequence was identified in a camel herd and its owner who died of severe respiratory disease [18]. Early results from an ongoing investigation in Qatar suggest that compared with others, those working closely with camels were at higher risk of MERS-CoV infection [19]. The route of transmission between humans and camels is however not established [19]. MERS-CoV RNA and anti-MERS-CoV antibodies have been isolated from camel milk [20] and camels have been shown to shed the virus through nasal secretions and feces [20]. The similarity between genetic sequences of the viruses isolated from humans and camels suggests direct transmission from camels to humans [14]. These findings are not direct evidence for a transmission route, and identifying direct or indirect transmission routes from camels to humans is crucial to stop disease transmission [19]. No consistent pattern of animal exposure has been observed among cases [14]. Clusters of human-to human transmission are well-documented, but are restricted to close contact within a family [21,22] or are healthcare-associated, where other patients and healthcare workers have been infected [23–25]. Healthcare clusters account for the majority of cases [19]. No community-wide outbreaks have been documented after extensive follow up of contacts of confirmed cases [25], and only two tertiary cases have been reported [19]. MERS-CoV was circulating in Saudi Arabia during several mass gathering events (Hajj pilgrimage in 2012 and 2013, Umrah in 2013), with no outbreaks among pilgrims reported during those events [26] in spite of extensive surveillance [27]. In light of the lack of sustained human-to-human transmission, in its latest risk assessment in October 2014 the European Centre for Disease Prevention and Control (ECDC) deemed the risk of human infection and sustained transmission in Europe to be low [2]. WHO has recommended that no specific screening or restriction to travel or trade are necessary [19]. In October 2014, the International Health Regulations Emergency Committee concluded that the conditions for a Public Health Emergency of International Concern (PHEIC) have not yet been met [28]. As the estimated reproductive number is below one [29], MERS-CoV does not appear to have pandemic potential.

In spite of the low risk of a global outbreak, more cases originating in the Middle East are expected. Epidemiological analysis of the distribution of cases over time suggests MERS-CoV could be seasonal, with an upsurge expected in the spring [28]. The spring peak corresponds to the end of camel calving season in Saudi Arabia [30], potentially suggesting a particular role for young camels in zoonotic transmission. This theory is supported by the higher frequency of infection and higher viral shedding in young camels compared with older ones [15,31–32]. The majority of reported cases have likely occurred through contact with another case, and only about a fourth of all cases are considered as primary [19]. Since the onset of the outbreak, the proportion of cases acquired from other humans has increased, which has been hypothesized by some to be caused by an increase in transmissibility [19]. There are several knowledge gaps surrounding the transmission of the virus. For example, there are reported cases with no recorded animal contact nor contact with other human cases [19]; and despite a low reproductive number, the virus still circulates in the human population more than 2 years after the initial case was reported. In contrast, the SARS coronavirus, which had a higher reproductive number, remained in the human population for less than a year [26]. Of further concern, rapid onset nosocomial outbreaks with a reproductive number over one have been recorded [23] and the virus has been isolated in the air of a camel barn where transmission occurred [33] raising the question of airborne transmission. Airborne transmission or sustained human-to-human transmission would profoundly alter the nature of the outbreak, with a much more severe impact and international spread.

In light of these uncertainties, a better understanding of the epidemiology of MERS-CoV combined with a holistic approach to surveillance and control in both humans and animals is crucial. We propose a multifaceted approach to preventing MERS-CoV spread combining research, surveillance, control, preparedness and risk communication.

## Future perspective

### • Research

There are still several question marks regarding how the virus transmits, where it originates and what the risk factors associated with infection are. Elucidating these questions will enable a better assessment of the risk the virus poses, as well as enabling the implementation of control strategies. One clear gap in the body of evidence surrounding MERS-CoV is the paucity of epidemiological studies aimed at identifying risk factors associated with MERS-CoV infection. In order to encourage such studies, WHO has prepared a protocol for case–control studies aimed at identifying such risk factors [34]. One hospital-based case–control study found few clinical predictors [35]. Up to November 2014, no case control or other epidemiological study to identify risk factors had been published. In June 2014, Saudi Arabia was noted as recruiting cases for such a study [13].

Serological studies, in both animals and humans can also help understand the epidemiology of infection and disease. Such studies have helped establish that the virus circulates in camels but not in other species [17], as well as suggested that the virus was not circulating in the Saudi population [36], thus leading to the conclusion that asymptomatic carriers were unlikely to play a major role in human-to-human transmission. Such studies have also highlighted the complexity of the epidemiology of MERS-CoV, as none of 226 Saudi camel slaughterhouse workers tested for MERS-CoV antibodies were seropositive [36], in spite of mounting evidence of the role of camels in spreading infection. Serological studies can also enable the comparison of seropositivity in different population groups and provide clues to populations at risk. WHO has made a seroprevalence study protocol available for research teams wishing to undertake such studies [37].

Finally, phylogenetic studies allow tracking of the movement of the virus, both across and within species. This type of study suggested that the virus jumped the species barrier from camels to humans [18]. Ongoing studies will help monitor future mutations and combined with epidemiological studies in humans, help assess the possibility of a change in the mode of transmission, which is particularly important to assess the sustainability of human-to-human transmission.

### • Surveillance at the animal, human & human-to-animal interface levels

As MERS-CoV is an emerging pathogen whose epidemiology is not yet fully understood, efficient surveillance is essential to monitor trends and distribution of the disease in terms of seasonality, age and sex distribution and countries of origin. Up to November 2014, MERS-CoV has been mainly sporadic [2]. Ongoing surveillance will allow detection of any increase in disease. Early detection of a disease peak, should one occur in the future, may enable early response, thus limiting spread and mitigating the impact. Furthermore, analyzing routine surveillance data for patterns of transmission may help to identify sustained human-to-human transmission, were it to occur. Early detection of such an event would be key to preventing a much larger outbreak. Although human infection is thought to have only originated in the Middle East, worldwide surveillance is important as camels harboring antibodies to MERS-CoV have been identified in other parts of the world, specifically Ethiopia, Nigeria, Kenya and Spain (Canary Islands) [38–40]. WHO has identified the detection of early human-to-human transmission and the identification of geographical risk areas as two key MERS-CoV surveillance objectives [40]. An integrated, one health approach to MERS-CoV surveillance involving communicable disease surveillance in humans and veterinary services is essential, ensuring systematic communication between the two, especially in ‘hotspots’ areas such as the Middle East and other areas with a significant camel population, especially where humans and camels are in close contact. Although camels have been identified as a reservoir for the virus, it is possible that there are other unidentified species asymptomatically hosting the virus. Animal surveillance should therefore not be restricted to camels.

### • Virological surveillance

In addition to epidemiological surveillance, continuous virological surveillance is necessary for monitoring viral RNA in newly identified cases, in order to identify new mutations that may occur. Early identification of mutations implies making viral genetic sequences available to research teams worldwide. Virological surveillance, in this outbreak as well as others therefore requires robust data sharing agreements. Following criticism about Saudi Arabia's initial reluctance to collaborate with international partners [41], many outbreaks sequences are publically available [42] and this trend must continue, ensuring information is shared as early as possible, to monitor the virus' evolution in a timely fashion.

### • Case definitions & case finding

Since the onset of the outbreak, WHO has revised its case definition for MERS-CoV six times, most recently in July 2014 [19], reflecting an evolving understanding of the disease. The current definition of probable cases focuses on respiratory symptoms and epidemiological links to either another confirmed case or travel or residence in the affected region [19]. While most reported MERS-CoV cases presented with severe respiratory infection, atypical presentations, such as gastrointestinal symptoms have occurred, particularly in immunocompromised individuals, creating a diagnostic challenge for clinicians [25]. As the understanding of the disease constantly progresses, such evidence should be considered when revising case definitions. There is a risk otherwise that infected individuals could be admitted to hospital without a suspicion of MERS-CoV infection, thus infecting unsuspecting healthcare workers and other patients. In May 2013, the French case definition was changed to include severe febrile clinical signs, including febrile diarrhea in immunocompromised persons or in those with chronic underlying conditions, returning from an at-risk country [25]. Understanding and monitoring MERS-CoV must extend beyond analyzing notified MERS-CoV cases. The ECDC advises that any person diagnosed with an acute respiratory infection and having been possibly exposed to MERS-CoV by visiting affected countries, working in an affected facility, having contact with dromedary camels or being part of an unexplained cluster of severe respiratory infections should be tested for MERS-CoV [2]. This approach ensures the detection of the emergence of MERS-CoV in a different part of the world.

### • Case management & infection control

Out of 72 MERS-CoV cases with a recorded source of infection which occurred between May 2013 and May 2014, 54 (75%) were acquired from another person [4], and 41 of the 54 (76%) were hospital acquired [4]. This suggests that, although there is no sustained human-to-human-transmission, the majority of cases are acquired from another person. In addition, a third of cases with an onset between May 2013 and May 2014 were healthcare workers [4]. Therefore, strict case management, and particularly strict infection control measures should be implemented in order to decrease the incidence of infection. WHO recommendations include the early triage and isolation of suspected cases, regular cleaning and disinfection of hospital rooms, restrictions on the number of visitors and healthcare workers in contact with suspected or confirmed cases, the use of hand hygiene, the use of personal protective equipment, including particulate respirators and face shields, the use of ventilated rooms and restricting patient movement [43]. WHO has also issued recommendations for care at home [44].

### • Continued risk assessment at local, regional & international level

MERS-CoV is still a poorly understood virus, with a rapidly changing epidemiology. In April 2014, the number of reported cases increased more than fourfold compared with the previous month, to more than 140. In contrast only two cases were reported between 10 July and 20 August 2014 [2]. These variations are not yet understood. In light of this, continuing and regular risk assessments are necessary in order to tailor advice locally and internationally to an evolving situation. ECDC's 12th risk assessment was released in October 2014 and WHO's latest was in June 2014. Regular updates are important, to ensure the situation is actively monitored, and are still useful to the international community even if the level of risk remains constant. The International Health Regulations emergency committee has had seven meetings on MERS-CoV between July 2013 and October 2014. Regularly reassessing whether MERS-CoV meets the conditions to be declared a Public Health Event of International Concern will enable the timely introduction of evidence-based prevention measures in the event the criteria are met. While these conditions are not met, regular statements from the International Health Regulations committee reinforce the message that travel restrictions are not required, thus potentially mitigating any economic impact caused by planned cancellation of trade or travel arrangements. Even as the risk remains constant, WHO's re-iteration of recommendations around good infection control and prevention practice, enhanced surveillance around religious pilgrimages in Saudi Arabia, harmonization of testing algorithms and veterinary surveillance [28] ensure that heightened awareness is maintained.

### • Risk communication in affected areas

A universal measure to decrease the impact of an outbreak is to tackle risky behavior. Although results from a case–control study identifying exposures associated with being an MERS-CoV case are still pending, drinking camel milk could be a risk factor for being a case. The consumption of raw camel milk is traditional in Arabic culture and sometimes among pilgrims visiting Saudi Arabia [2]. Risk communication around the consumption of camel milk, taking into consideration the cultural significance of such a behavior in Arab countries and identifying interventions that are both culturally appropriate and reduce the risk of transmission, will help decrease disease incidence.

### • Preparedness planning

The best-case scenario for MERS-CoV is for human-to-human transmission to remain unsustained. This would maintain the status quo where cases occur sporadically with occasional peaks rapidly subsiding. A better understanding of the animal-to-human interface could even lead to control measures resulting in a decrease in transmission to humans. Even in the context of limited human-to-human transmission, nosocomial MERS-CoV outbreaks are not rare events and have been reported in Saudi Arabia [23], Qatar, Jordan [45] the United Arab emirates [46], the UK [47] and France [25]. In order to prevent such events, hospitals should prepare for the possibility of receiving MERS-CoV patients and have a plan in place to ensure the virus does not spread to other patients or to healthcare workers. The US Center for Disease Control and Prevention have designed preparedness checklist for healthcare facilities [48] and frontline healthcare providers [49] that help prevent the introduction of a patient with MERS-CoV in a healthcare facility without a diagnosis. Other countries, especially those with a high volume of travel to and from the Middle East, should have similar preparedness checklists in place.

The worst-case scenario for MERS-CoV is a mutation in the virus that would enable sustained human-to-human transmission through transmission via respiratory droplets, in a similar fashion to SARS transmission. The two viruses are phylogenetically distinct [9] and produce different clinical and pathological pictures [50] and there is no evidence to suggest MERS-CoV can or will produce a SARS-type outbreak. In spite of a very low risk of a SARS-like scenario with MERS-CoV, useful lessons from managing the SARS outbreak at the local, national and international level can be applied to MERS-CoV, in particular: building local public health surveillance capacity to enable rapid detection of the disease, high quality data collection and systematic and timely data reporting, integrating disease-specific preparedness into wider local preparedness planning, providing resources, training and expertise to local hospitals to strengthen infection control practice and developing agreements for specimen and data sharing [51].

Considering the low risk of sustained human-to-human transmission, and therefore the low risk of large community outbreaks, detailed community preparedness planning, including isolation and quarantine, are not necessary specifically for MERS-CoV in light of current evidence. However, should the epidemiological situation change, translating SARS preparedness planning to the MERS-CoV context would be a rapid way to ensure the impact of the outbreak is mitigated.

## Conclusion

MERS-CoV is a novel virus associated with high mortality. Current knowledge about the virus indicates a low potential for sustained human-to-human transmission, suggesting a low potential for MERS-CoV to become a substantial global threat. Up to November 2014, all cases originated in the Middle East or were epidemiologically linked to cases from the Middle East. Camels appear to be the main animal reservoir, although direct human-to-human transmission in healthcare settings account for more cases than direct contact with camels. There are still several important knowledge gaps around the disease, and as such different scenarios, including sustained human-to-human transmission, should not be completely discarded.

In order to prevent global spread, a combination of local, regional and global measures are required. Locally, the ability to detect and report cases in a timely manner and to apply the highest standards of infection control will limit the spread. Regionally, and particularly in the Middle East, culturally sensitive risk communication, evidence-based travel recommendations and sensible preparedness will both limit the spread and mitigate the impact in case of a change in the epidemiology. Globally, the ability to address knowledge gaps, through descriptive and analytical epidemiology, phylogenetic studies and serological surveys will help better understand the disease. This implies access to information from affected countries, including genetic and epidemiological data. This information will also inform a continuous risk assessment process.

Overall, these measures are already in place and the risk of a large-scale outbreak is currently very low. However prior to the Ebola outbreak still affecting West Africa as of November 2014, Ebola was considered a disease affecting remote rural communities, with no or low potential for large-scale spread. This must serve as a sobering reminder that the epidemiology of a disease can change in a different environment. Preparedness, based on continued risk assessments, must be embedded in the management of the outbreak.

Overall, all the tools to prevent spread on a larger scale exist: high quality epidemiological and virological surveillance, clinical management and infection control protocols and preparedness tools. The key to success relies on the application of these tools, the sharing of information globally in a transparent manner and maintaining and using global communication channels, such as the International Health Regulations if any further spread is suspected.

**EXECUTIVE SUMMARY**

**MERS-CoV emerged in 2012 & to date has been a low transmissibility, high mortality pathogen**

- Middle East respiratory syndrome coronavirus (MERS-CoV) has caused a total of 935 confirmed human infections with 371 deaths.
- All cases have occurred in individuals living in or traveling to the Middle East, or who had contact with a person who had traveled to the Middle East.
- Most cases are secondary, either healthcare acquired or acquired through close family contact.
- No sustained human-to-human transmission.
- Increasing evidence for camels being the reservoir, with regular re-introduction in the human population.
- Risk of global spread is low.

**Questions about MERS-CoV epidemiology remain unanswered**

- Transmission mechanism from animals to humans remain unclear.
- Persistence in human population in spite of low reproductive number not fully explained.
- Potential for rapid onset nosocomial outbreaks established.
- Possibility of airborne transmission raised.

**Uncertainty about MERS-CoV warrants a multifaceted approach to risk assessment & surveillance**

- Research: for a better understanding of the source/reservoir of the virus, mode of transmission and risk factors for infection:
  - Case–control studies;
  - Serological studies;
  - Phylogenetic studies.

- Surveillance at the animal, human and human-to-animal interface levels:
  - One health approach;
  - Epidemiological and virological.

- Case definitions:
  - Should be dynamic to reflect progress on understanding the disease.

- Case finding:
  - To ensure a full epidemiological picture;
  - To ensure the emergence of the disease in another part of the world.

- Case management and infection control:
  - To decrease transmission in healthcare and community settings.

- Continued risk assessment at local, regional and international level:
  - Continued International Health Regulation assessments;
  - Travel recommendations.

- Risk communication in affected areas:
  - Culturally sensitive messages to reduce risk of infection.

- Preparedness:
  - Consider the worst case scenario;
  - Lessons from SARS.

**Conclusion**

- The risk of global spread of MERS-CoV is low.
- Mechanism of transmissions are not fully understood.
- Better understanding of disease will be key to further assess risk.
- Combination of research, surveillance, infection control around cases, risk communication and preparedness across local, regional and global will help better quantify the risk posed by MERS-CoV and better prepare.
- Tools to prevent further spread are all available.
